# Functional exercise capacity, strength, balance and motion reaction time in Barth syndrome

**DOI:** 10.1186/s13023-019-1006-8

**Published:** 2019-02-11

**Authors:** Brittany Hornby, Rebecca McClellan, Lucy Buckley, Kimberley Carson, Tiffany Gooding, Hilary J. Vernon

**Affiliations:** 1 Department of Physical Therapy, Kennedy Krieger Institute, Baltimore, MD USA; 2 Department of Neurogenetics, Kennedy Krieger Institute, Baltimore, MD USA; 3 Bristol Royal Hospital for Children, Bristol, UK; 4 Child Development and Rehabilitation Center, Johns Hopkins All Children’s Hospital, St. Petersburg, Florida USA; 5 Department of Pediatrics, McKusick-Nathans Institute of Genetic Medicine, Johns Hopkins University, Baltimore, MD USA

**Keywords:** Barth syndrome, 6-min walk test, Myopathy, SWAY balance

## Abstract

**Background:**

Barth syndrome (BTHS) is an X-linked disorder caused by defects in *TAZ* with key clinical features including cardiomyopathy, neutropenia and skeletal myopathy. In order to gain a better understanding of the range of clinical features, identify targets for monitoring, and increase knowledge of natural history of the disease, we conducted muscle strength testing, functional exercise capacity testing, physical activity assessment, balance assessment and motion reaction time testing in 33 affected individuals and 14 controls. We analyzed data points to provide a cross-sectional quantitative spectrum of disease characteristics. We also compared these data points to the matched data points collected two years prior to provide insight into effects of BTHS over time.

**Results:**

In comparison to controls, pediatric subjects with BTHS present with significantly impaired balance and motion reaction time while adult subjects with BTHS present with significantly impaired motion reaction time. In comparison to controls, subjects with BTHS presented with decreased functional exercise capacity (assessed via 6 MWT), knee extensor strength (both assessed via handheld dynamometry and five times sit-to-stand (5 TSTS)), and self-reported physical activity. Comparison of functional exercise capacity, knee extensor strength and self-reported physical activity from identical cohorts in 2014 and 2016 BTHS showed that the deficits in 6 MWT do not change significantly over the 2 year time span.

**Conclusion:**

In this comprehensive assessment of musculoskeletal parameters in a cross-section of individuals with BTHS, we uncovered deficits in motion reaction time and balance, which were previously not known to be abnormal in in BTHS. We also confirmed results of our previous study showing that pediatric and adult subjects with BTHS have decreased functional exercise capacity, knee extensor strength, and physical activity in comparison to controls, r, verifying the importance of including these measures as part of the regular clinical assessment in individuals with BTHS, as well as introducing 5 TSTS as an additional testing parameter. Perhaps most importantly, we demonstrated that 6 MWT results do not significantly vary in pediatric and adult cohorts with BTHS over a 2-year period, supporting this as a reliable quantitative measure of therapeutic outcomes in clinical studies and for clinical monitoring.

## Background

BTHS (3-methylglutaconic aciduria type II, MIM 300394) is a rare X-linked disorder with an estimated prevalence of 1/300,000–400,000 live births characterized by cardiomyopathy, neutropenia, growth abnormalities, and skeletal myopathy, among other features [1]. Skeletal myopathy in BTHS predominantly affects the proximal musculature and has a significant effect on exercise tolerance and quality of life [2]. The myopathy often leads to developmental motor delay, with about 72% of affected children having a delay in walking [3]. Patients with BTHS present with exercise intolerance that is thought to be due to both cardiac impairment and decreased skeletal muscle oxygen utilization [4].

Daily functional activities, such as ambulation, stair climbing, and transfers, require a combination of muscular strength, endurance, and balance. In our prior cross-sectional phenotyping study in with BTHS, we found that affected individuals had significantly impaired functional exercise capacity as measured by 6MWT, knee and hip flexor and extensor strength, and reduced daily activity. In the present study, we expanded upon the musculoskeletal assessments performed in our previous study by including further functional testing in areas known to be affected by hip and knee flexor weakness [5, 6], and with high importance for quality of life and daily functioning (sit to stand testing (5 TSTS), quantitative balance assessment) and evaluated progression of musculoskeletal capacity in a subset of individuals over 2 years [2]. Knowledge of strength, functional exercise capacity, and balance will facilitate standardization of clinical assessments in BTHS, thereby allowing for improved ability to assess ongoing patient  status and quantitatively evaluate response to treatment.

## Results

### Patient cohort

The study group included a total of 33 males with BTHS ranging in age from 6 years to 34 years (average age 16.2 ± 7.7, median age 15) and 14 controls ranging in age from 6 years to 30 years (average age 14.9 ± 6.8, median age 12). The study population was divided into a pediatric cohort, age 6–19 years (BTHS *n* = 24, Control *n* = 10), and an adult cohort > 20 years (BTHS *n* = 9, Control *n* = 4) for the purposes of data analysis, and based on available published normative data.

Average height of the pediatric BTHS participants did not differ significantly from the control population (144.5 cm +/− 24.67 SD (median 143) vs. 148.8 cm +/− 17.18 SD (median 145.5) respectively), and both were within the normal range based on normative population data provided by the CDC [7]. Average weight of the pediatric BTHS cohort (6-19y) did not differ significantly from the control population (37.77 kg +/− 15.58 SD (median 35) vs. 39.9+/− 11.80 SD, (median 42.5) respectively), and both were within the normal range based on normative population data provided by the CDC. Average height of the adult BTHS participants did not differ significantly from the control population (175.9 cm +/− 8.39 SD (median 177) vs. 180 cm +/− 2.83 SD (median 180) respectively), and both were within the normal range based on normative population data provided by the CDC. Average weight of the adult BTHS cohort was significantly less than the control population (66.22 kg +/− 11.62 SD (median 68) vs. 113.5+/− 14.85 SD) respectively. The adult BTHS cohort weighed less and the control population weighed more compared to normative population data provided by the CDC (50th percentile 80.7 kg). The adult control data is somewhat limited, as height and weight was missing for 2 participants.

Baseline vital signs taken prior to clinical testing revealed that none of the participants had hypertension, all had heart rates in the normal range, and all had normal oxygen saturations. 18 subjects with BTHS participated in our previous study in 2014 and this current study.

### The 6 MWT

**Fig. 1:**
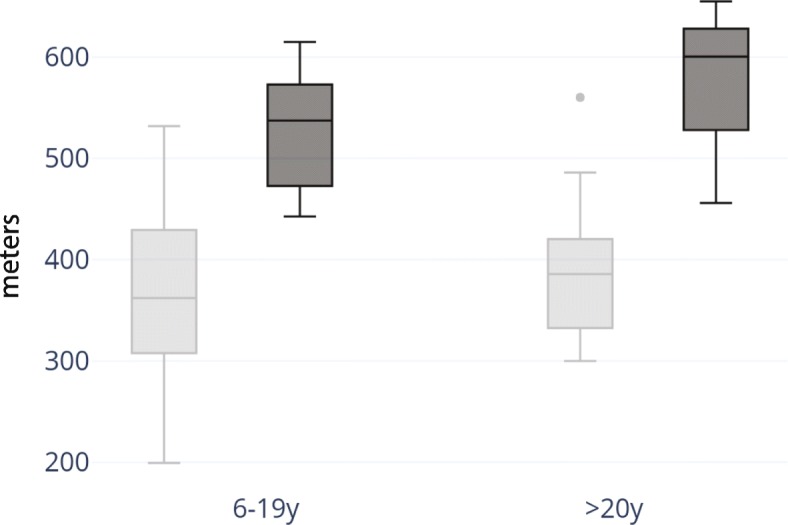
Performance on 6 MWT on a 15-m course. Barth 6 MWT distance (m) for pediatric subjects age 6–19 years of age (BTHS *n* = 24, control *n* = 10) and for adult subjects 20–34 years of age (BTHS *n* = 9, control *n* = 4). On average, subjects aged 6–19 walked 67% of controls on the 6 MWT and subjects aged 20–34 walked 68% of controls on the 6 MWT. BTHS cohorts are represented in light gray, and control cohorts are represented in dark gray

**Fig. 2:**
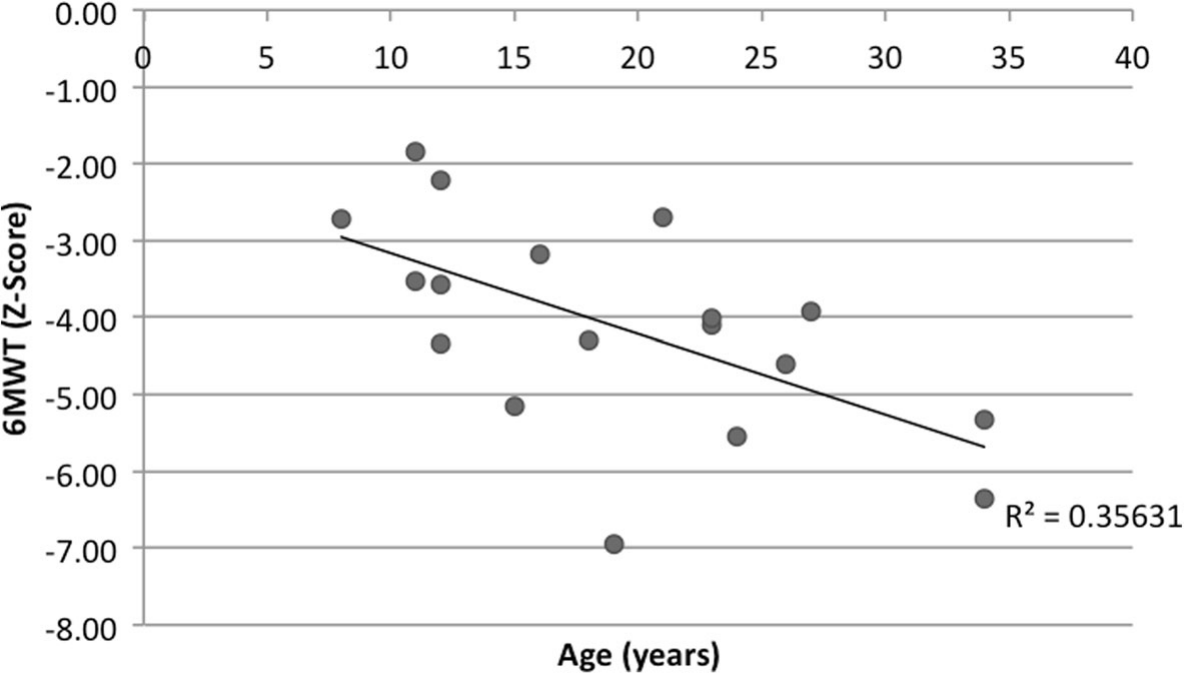
6MWT and population averages. 6MWT in BTHS subjects (*n* = 33) and SD from height-adjusted average. When comparing 6 MWT distance to reported population averages, only 1 BTHS subject walked within the normal range (±2SD)

Functional exercise capacity was measured in 33 participants with BTHS ages 6 years to 34 years and 14 control participants ages 6 years to 30 years via the 6 MWT. The 6 MWT results were compared to those of controls and also to SD from the height-adjusted average of reported normal population controls (Figs. 1 and 2) [8–10]. Pediatric and adult controls walked significantly farther during the 6 MWT in comparison to subjects with BTHS (Age 6–19: *p* = 7.12 × 10^− 7^, Age 20–34: *p* = .01).The pediatric BTHS cohort walked 359.2 m +/-86SD (median 364.3) whereas the pediatric control cohort walked 533.2 m +/− 62 SD (median 555). The adult BTHS cohort walked 392.7 m +/− 83 SD (median 386) whereas the adult control cohort walked 578 m +/− 85.3SD (median 600.6).

Pediatric subjects with BTHS present with significantly increased fatigue pre- (*p* = .0013) and post- (*p* = .04) 6 MWT and dyspnea post- (*p* = .03) 6 MWT in comparison to controls (Table 1). Adults with BTHS presented with significantly increased dyspnea pos-t (*p* = .01) 6 MWT in comparison to controls. Individuals with BTHS present with significantly increased heart rate pre- 6 MWT (*p =* .006) in comparison to controls (Table 2).

**Table 1:** Average Fatigue and dyspnea pre/post 6 MWT using the Borg Scale for BTHS subjects and controls

Modified Borg Measure	Barth Age 6–19 years (*n* = 24)	Control 6–19 (*n* = 10)	Barth 20+ years (*n* = 9)	Control 20+ years (*n* = 4)
Fatigue Pre 6 MWT	1.52 ± 1.56^a^	0.3 ± 0.42	1.28 ± 1.25	0.25 ± 0.5
Fatigue Post 6 MWT	3.35 ± 2.69^a^	1.5 ± 2.05	3.28 ± 2.14	1.75 ± 2.22
Dyspnea Pre 6 MWT	0.63 ± 1.19	0.4 ± 0.66	0.33 ± 0.66	0.75 ± 0.96
Dyspnea Post 6 MWT	2.85 ± 2.38^a^	1.4 ± 1.33	2.83 ± 1.27^a^	2.13 ± 1.18

^a^ significant differences (*P* value of < 0.05)

**Table 2:** Average Heart Rate Pre and Post 6 MWT for BTHS subjects and controls

	Heart Rate Pre (bpm)	Heart Rate Post (bpm)	Change (Post-Pre)
Barth Age 6–34 (*n* = 24)	92 ± 16^a^	108 ± 25	16 ± 18
Control Age 10–30 (*n* = 11)	80 ± 9	100 ± 23	20 ± 19

^a^ significant differences, (*P* value of < 0.05). 7 subjects with BTHS and 3 controls excluded from analysis secondary to technical difficulties with pulse oximeter

### Strength values

**Fig. 3:**
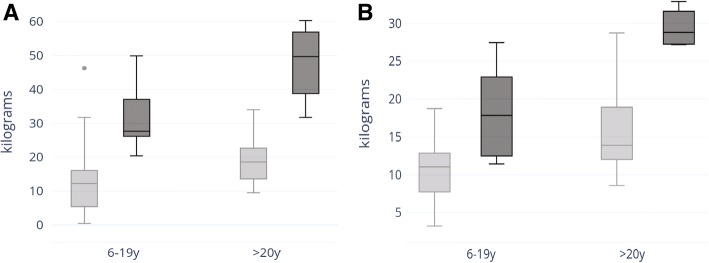
Strength testing in BTHS subjects. Range, median, upper and lower quartile of grip strength (**a**) and knee extensor strength (**b**) in kilograms for subjects 6–19 years of age (BTHS *n* = 24, control *n* = 10) and for subjects 20–34 years of age (BTHS *n* = 9, control *n* = 4). BTHS cohorts are represented in light gray, and control cohorts are represented in dark gray

Strength testing was completed in 33 individuals with BTHS (24 children ages 6–19 years, 9 adults > 20 years) and in 14 controls (10 children ages 6–19 years, 4 adults > 20 years). Age cohorts were selected because of available published population normal values [11, 12]. Knee extensor strength measurements obtained via handheld dynamometry (HHD) for BTHS subjects were compared to control subjects. Given that values for right and left knee extensors were very similar for all subjects, only right knee extensor strength values are provided (Fig. 3, Table 3). On average, subjects with BTHS aged 6–19 have knee extensor strength that is 57% of controls and subjects with BTHS aged 20–34 have knee extensor strength that is 54% of controls. Controls present with significantly increased knee extensor strength in comparison to pediatric (*p = .*0017) and adult (*p =* .005) subjects with BTHS. In pediatric subjects with BTHS, there is a strong correlation between age and knee extensor strength (*r* = .59). In adult subjects with BTHS, there is a strong negative correlation between age and knee extensor strength (*r* = −.46). In pediatric and adult controls, there was a very strong positive correlation between age and knee extensor strength (pediatric *r* = .79, adults *r* = .88). This finding of a strong, positive correlation between age and strength in the pediatric controls is consistent with published reference values [13, 14].

**Table 3:** Average force of right knee extensors, right grip strenth, and 5 TSTS in subjects with BTHS and controls

	Knee Extensor Strength (kg)	Grip Strength (kg)	5 TSTS (seconds)
BTHS Age 6–19 years (*n* = 24)	10.57 ± 3.77^a^	13.00 ± 10.64	9.52 ± 2.51^a^
Control Age 6–19 years (*n* = 10)	18.57 ± 5.85	19.55 ± 7.61	6.23 ± 1.00
BTHS Age 20+ years (*n* = 9)	15.98 ± 5.96^a^	32.11 ± 19.14	10.74 ± 3.88^a^
Control Age 20+ years (*n* = 4)	29.42 ± 6.01	47.85 ± 12.27	6.26 ± 2.31

^a^ significant differences (*P* value of < 0.05)

Grip strength measurements obtained via HHD for subjects with BTHS were compared to control subjects. Given that values for right and left grip strength were very similar for all subjects, only right grip strength values are provided (Fig. 3, Table 3). On average, BTHS subjects aged 6–19 have grip strength that is 67% of controls, and BTHS subjects age 20+ present with grip strength that is 67% of controls. In the entire BTHS cohort (aged 6–34, *n* = 33) and entire control cohort (age 6–30, *n* = 14), there was a very strong correlation between age and grip strength (BTHS: *r* = .76, control: *r* = .88).

### 5 time sit to stand (5 TSTS)

**Fig. 4:**
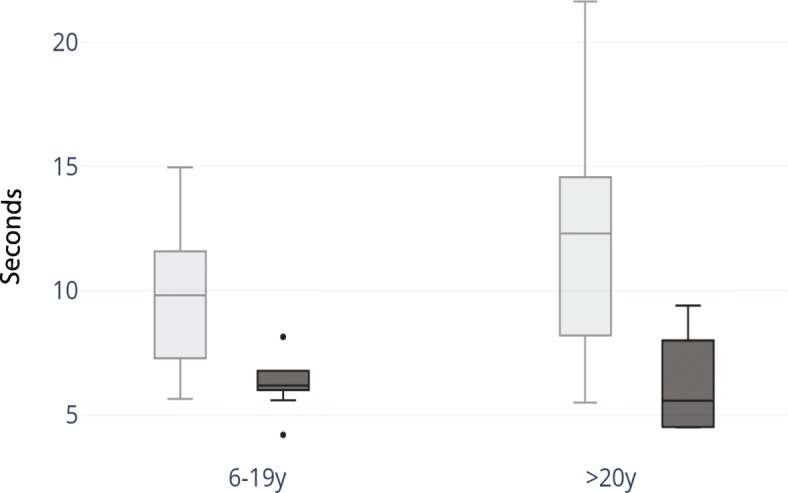
5 TSTS in subjects with BTHS. Range, median, upper and lower quartile of 5 TSTS for subjects 6–19 years of age (BTHS *n* = 24, control *n* = 10) and for subjects 20–34 years of age (BTHS *n* = 7, control *n* = 4). BTHS cohorts are represented in light gray, and control cohorts are represented in dark gray

5 TSTS values (in seconds) were compared between subjects with BTHS (*n* = 31) and controls (n = 14) (Table 3, Fig. 4). One adult with BTHS required use of a single hand and one adult with BTHS required use of bilateral hands to complete the test, and therefore their values were removed from data analysis. Both pediatric and adult subjects with BTHS required significantly more time to complete the 5 TSTS in comparison to controls (Age 6–19: *p* = 3.92 × 10^− 6^; Age 20+: *p* = .04). On average, pediatric controls were able to complete the 5TSTS in 65% of the time of pediatric subjects with BTHS and adult controls were able to complete the 5 TSTS in 58% of the time of adults with BTHS.

### Physical activity

*Subjects age 6–14*. In total, 16 subjects with BTHS and 9 controls between ages 6 and 14 completed the Physical Activity Questionnaire for Children (PAQ-C). The average PAQ-C activity score calculated for subjects with BTHS was 2.49 ± 0.67, and the average PAQ-C activity score calculated for controls was 3.41 ± 0.80. This indicates that, on average, subjects with BTHS age 6–14 demonstrate a low to moderate level of physical activity while control subjects demonstrate a moderate to high level of physical activity. Control subjects age 6–14 demonstrate significantly more physical activity in comparison to subjects with BTHS (*p* = .01). There was a negligible correlation between the average PAQ-C activity score and 6 MWT for subjects with BTHS (*r* = .18) and positive correlation for controls (*r* = .33). There was a positive correlation between the average activity reported on the PAQ-C and 6 MWT for both subjects with BTHS (*r* = .32) and controls (*r* = .42).

*Subjects age 15–34*. In total, 12 subjects with BTHS between the ages of 15 and 34 completed the International Physical Activity Questionnaire (IPAQ). In total, 5 control subjects between the ages of 15 and 30 completed the IPAQ. Of the 12 questionnaires completed by subjects with BTHS that were utilized in analysis, 3 subjects (25%) indicated a low physical activity level, 1 subject (8.3%) indicated a moderate activity level and 8 subjects (66.7%) indicated a high activity level. Of the 5 questionnaires completed by controls, 5 (100%) indicated a high level of physical activity. In comparison to subjects with BTHS, on average, controls participate in vigorous activity more days per week (*p* = .005). In subjects with BTHS and controls, there was a moderate correlation between days per week spent walking and 6 MWT distance (Barth: *r* = .37, Control: *r* = .30). In controls, there was a strong, negative correlation between average amount of time spent sitting per day and distance walked on the 6 MWT (*r* = −.65). In subjects with BTHS there was a negligible correlation between average amount of time spent sitting per day and distance walked on the 6 MWT (*r* = .14). In subjects with BTHS, there was a negative correlation between days spent participating in moderate activity and 5 TSTS time (*r* = −.47). In controls, there was a very strong, negative correlation between days spent participating in moderate activity and 5 TSTS time (*r* = −.86).

### Balance

Of the subjects with BTHS aged 6 to 34, 33 subjects completed the balance and motion reaction assessment using the SWAY Balance application (SWAY Medical, Tulsa, Oklahoma). Of controls aged 6 to 30, 14/14 completed the balance and motion reaction assessment using SWAY Balance. One 6 year old subject with BTHS was only able to complete 1 trial secondary to fatigue. All controls were able to complete 3 trials.

Average baseline scores across all SWAY Balance categories (combined, motion reaction time, balance, balance left and balance right) in subjects with BTHS aged 6–19 are significantly decreased when compared to controls (combined: *p* = .02, motion reaction time: *p* = .03, balance: *p* = .01, balance left: *p* = 0.03, balance right: *p* = 0.02). Subjects with BTHS Age 20+ demonstrate significantly increased motion reaction time in comparison to controls (*p* = .04).

**Fig. 5:**
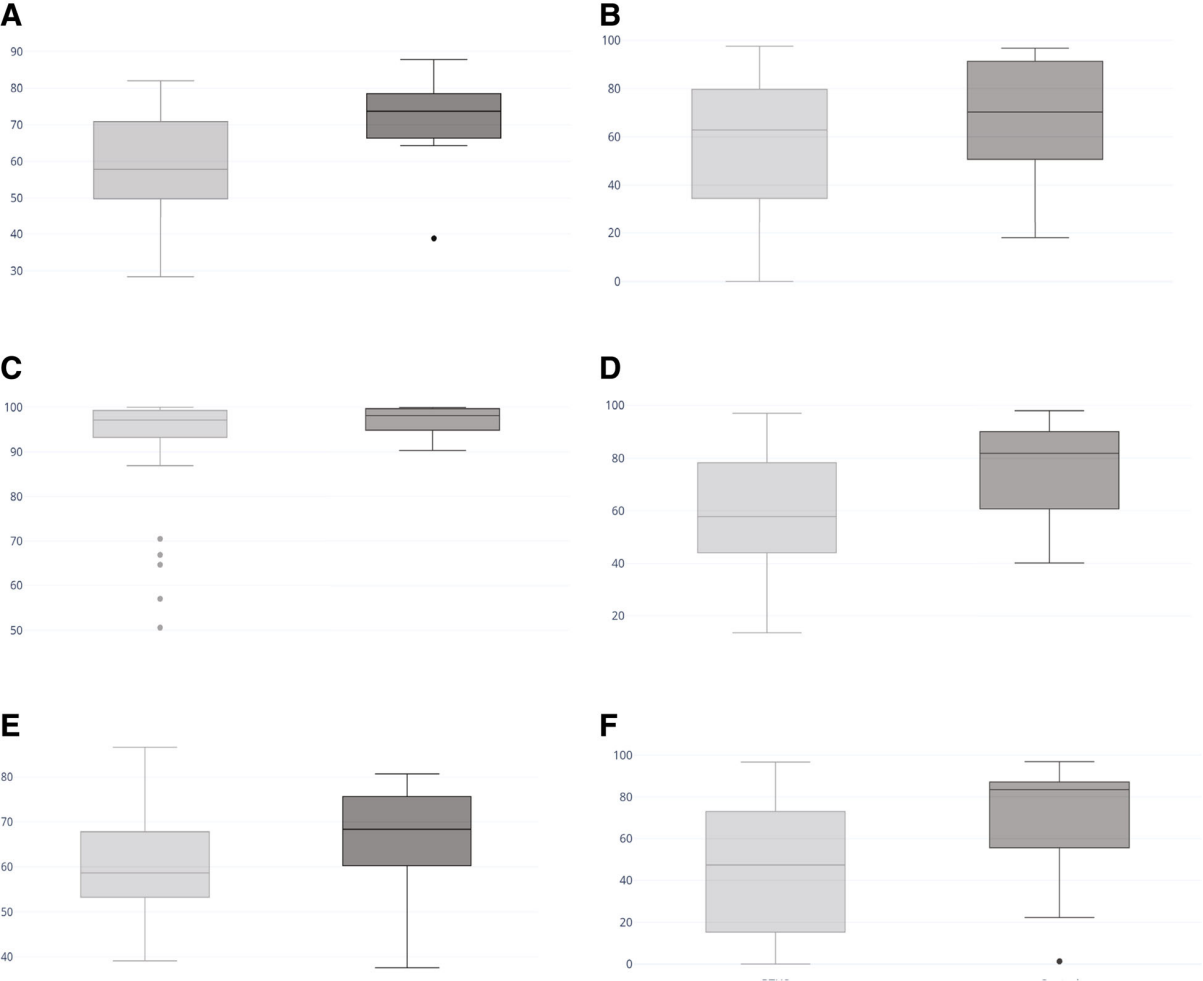
SWAY Balance scores in BTHS. Comparison of SWAY Average baseline score for (**a**) Combined score, (**b**) Feet Together EC, (**c**) Tandem Right EC, (**d**) Balance, (**e**) Motion reaction time, and (**f**) Single Leg Right EC for subjects aged 6–34 with BTHS (*n* = 33) and control subjects age 6–30 (*n* = 14). BTHS cohorts are represented in light gray, and control cohorts are represented in dark gray

SWAY scores for all subjects with BTHS (aged 6 to 34) were significantly lower compared to control subjects (aged 6 to 30), in the following categories (Fig. 5): Combined score (*P* = 0.003), Balance (*p* = 0.004), Feet Together EC (*p* = 0.03), Single Leg Right EO (*p* = 0.01).

### Total cohort analysis, comparing prior and current data

We compared data from the total BTHS subject cohorts participating in our prior and current studies. During our previous study [2], we found a negative correlation between baseline fatigue measured by the Modified Borg Scale and 6 MWT distance (m) in both pediatric and adult subjects with BTHS (*r* = −.03, *r* = −.24). Those results were replicated during this study (pediatric subjects *r* = −.26, adult subjects *r* = −.34). Based on these results, it appears that if an individual with BTHS reports increased fatigue at baseline, it will negatively impact the distance he is able to complete on the 6 MWT.

During our previous study we found that when normalized for body weight, pediatric subjects with BTHS presented with significantly greater knee extensor strength than adults with BTHS. In this study, by contrast, adults presented with a slightly higher knee extensor strength on average than pediatric subjects (35.24% ± 10.03 vs. 31.13% ± 10.39) (*p* = 0.14).

When comparing the 16 IPAQ questionnaires completed in 2014 by subjects with BTHS to the 12 completed during the 2016 conference we found that in 2014, 43.75% of subjects reported a high level of physical activity while 66.7% reported a high level of physical activity in 2016. In 2014, there was a strong positive correlation between days per week spent walking and 6 MWT (r = .53). In 2016, there was a moderate positive correlation between days per week spent walking and 6 MWT (r = .37).

### Longitudinal data analysis with common participant cohort

**Fig. 6:**
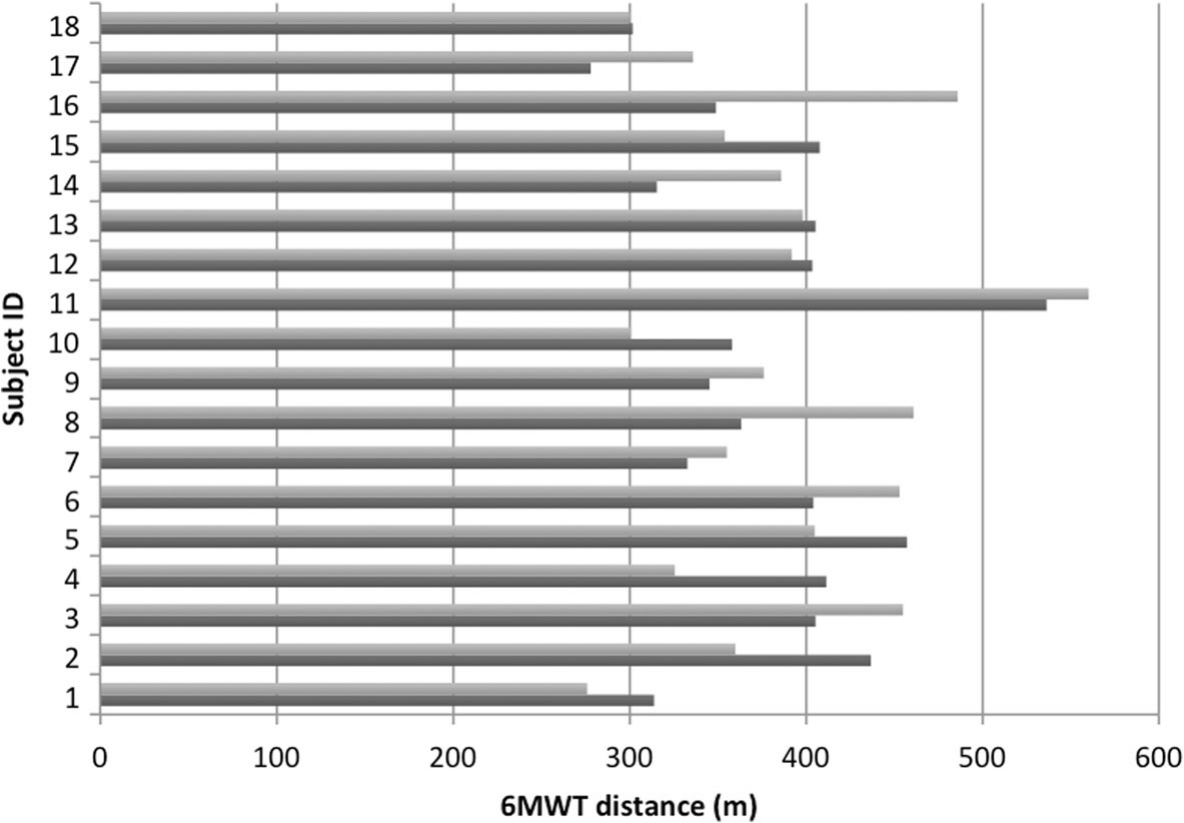
2-year 6MWT follow up. Comparison of 6 MWT distance (m) of common participants between 2014 (dark gray) and 2016 (light gray) (*n* = 18)

18 subjects with BTHS participated in our previous study and this current study. When looking at the pediatric cohort (subjects < 20 years based on age in 2014, *n* = 11), across the two data collection time points, we saw a statistically significant increase in fatigue reported post 6 MWT (*p* = .03) and raw left knee extensor strength (*p* = .04). We did not see a statistically significant difference between any common data points for subjects > 20 years old (*n* = 7). Perhaps most strikingly, we did not see a statistically significant difference between 6MWT distance across the cohort of 18 subjects (Fig. 6). In 2014, the average distance walked was 379 ± 63 m (SD) and in 2016 the average distance walked was 387.8 m ± 71 m (SD).

When examining the 6MWT performance within individuals, rather than an entire cohort, several individuals had significant changes in their distance walked. However, for the majority of these subjects with the greatest changes, major health events in the 2-year interim likely account for these differences.

## Discussion

Developing a thorough understanding of the musculoskeletal phenotype of individuals with Barth Syndrome will allow clinicians to appropriately evaluate ongoing disease symptoms, monitor response to ongoing therapies, and establish tools for evaluation of new therapeutics. In this study, we assessed strength, functional exercise capacity, and balance using tools that are readily available in most clinical settings, and are feasible to perform at regularly scheduled appointments. We further validated the utility of several of these clinical tools with a 2-year follow up study. These tools evaluate areas of known deficiency in BTHS (lower extremity strength, exercise capacity) and novel areas of deficiency uncovered in this present study (balance, motion reaction time).

We found, for the first time, that pediatric subjects with BTHS demonstrate significantly decreased balance and adult subjects with BTHS demonstrate significantly decreased motion reaction time, using a new assessment tool: the SWAY Balance. In evaluating overall trends, we found that motion reaction time testing in both patients with BTHS and controls revealed a trend of an improvement following adolescence, a peak in young adulthood and a slow decline with age. Previous research of motion reaction in other applications has shown a similar phenomenon [15].

There are several considerations to using the SWAY Balance system. We observed significant variability in the 3 serial balance measurements in both patients and controls, which is likely due to postural fatigue. This is not unexpected, as previous studies have demonstrated that muscle fatigue can alter postural control [16]. In the future, we recommend performing 2 trials of the SWAY Balance protocol at a maximum to avoid fatigue from affecting test results in subjects with BTHS. Additionally, in our current study, we only manipulated the visual system (EC and EO) and motion reaction time. Future studies that manipulate the vestibular and proprioceptive systems would be beneficial to determine if there is one system that most affects balance in the BTHS population [17], although these are unlikely to be major contributors as there is limited CNS involvement in this condition.

Deficits in functional exercise capacity via other methodologies have been previously demonstrated in BTHS [18], and we re-demonstrated that 6MWT test is abnormal in almost all individuals with BTHS tested [2]. Prior to this study, a retrospective analysis of BTHS patients who participated in a variable number of exercise physiology tests found that VO_2peak_ did not longitudinally change over time (average 5 years), thus arriving at a similar conclusion that exercise tolerance is relatively stable over time. However such studies are not feasible to be performed on a regular basis in most clinics due to equipment availability, complexity of testing, cost, and insurance coverage and therefore have limited practical clinical utility [18]. Thus, 6MWT represents a feasible tool for regular, longitudinal assessment of functional exercise capacity.

We further showed that 6MWT results do not significantly vary in pediatric and adult cohorts with BTHS over a 2-year period. However, several individuals had significant changes in the distance walked on the 6MWT. The majority of these subjects had major health events in the 2-year interim, which likely account for these differences Of the individuals with a significant improvement: Participant 16, who had a 137 m increase in 6 min walk test (shift in Z-score corrected for height from − 6.5 to − 3.9), underwent a cardiac transplant in the interim. While we have previously shown that cardiac function does not directly correlate to 6MWT in this population, multiple mitigating health factors surrounding the transplantation have an important potential role, such as introduction of new medications including steroids, improved overall health condition, and increased daily activities. Participant 8 had a 98 m improvement on his 6-min walk test (shift in Z-score corrected for height from − 4.5 to − 1.3). At the time of the 2014 study, he was recovering from a rib contusion, and was healthy in 2016. Of note, an interim 6MWT obtained in our clinic in 2014 was 512 m, confirming that the 2014 meeting data was likely aberrant due to the injury.

Of the individuals with a worsening of distance walked on 6MWT: Participant 4 had an 86-m decrease in 6MWT (shift in Z-score for distance corrected for height from − 2.96 to − 4.5). He was noted to be feeling unusually tired from his busy activities during the test day in 2016 (as noted in his pre-test Borg fatigue score), likely resulting in an aberrant outcome. Further in support of this, 6MWT values obtained in our clinic in 2015 and 2017 were in close agreement with the 2014 value (436 m and 457 m respectively). Participant 2 had a 77-m decrease in 6MWT performance, and unfortunately at this time the participant was suffering from a persistent health decline in multiple domains that preceded the meeting date in 2016 and continued after the meeting.

The only individual with a major change in 6MWT distance and no known significant interim health event, is participant 14, with a 70 m improvement. This is not an individual who regularly is seen in our outpatient clinic, so it is not known if one of these values was aberrant due to potentially mitigating circumstances, or this represents a true improvement over time. These findings emphasize the importance of multiple measurements over time in order to identify aberrant values, as well as consideration of major interim health events as contributors to 6MWT performance. In total, however, the two serial studies show that individuals with BTHS never perform within the expected range for height and age, the majority of individuals do not have significant changes in their 6MWT performance over 2 years, and the cohort performance for 6MWT is reliably in the 350–400 m range. Additionally, the 2 serial studies suggest a decline in 6MWT performance with increasing age, however at this time it is not clear if this represents a true age related decline or a phenomenon that appears due to an “adjustment advantage” for height in childhood. Longer-term studies in individual participants will help to bear this out.

### Study limitations

One drawback of this study is the small number of controls included in this study (*n* = 14) due to availability of control subjects at the study location. We complemented this small control number by also comparing to published population normative values and to previous study data.

In this current study we did not perform echocardiograms, and therefore cannot completely rule out the role of cardiac function in 6MWT performance. However, in our prior published investigation, we found no correlation between distance walked on the 6MWT and cardiac function. This is likely due to the fact that most individuals have cardiac function in the normal or near-normal range with medical treatment, and therefore the limitations of the significant skeletal muscle disease outweighs the contribution of mildly abnormal cardiac function.

Future longitudinal natural history studies regarding clinical measures of strength, functional exercise capacity and balance are needed to better clarify long-term evolution of symptoms with increasing age in individual patients. These studies are ongoing in our group.

## Conclusions

In this study, we assessed strength, functional exercise capacity, and balance using tools that are readily available in most clinical settings, and are feasible to perform at regularly scheduled appointments. These tools include established methodologies (HHD, 6MWT) to evaluate areas of known deficiency in BTHS (lower extremity strength, exercise capacity), and new tools (SWAY Balance) to evaluate novel areas of deficiency uncovered in this present study (balance, motion reaction time). We further validated the utility of several of these clinical tools with a 2-year follow up study. Importantly, we showed that the 6 MWT does not vary significantly in a cohort of individuals with BTHS over 2 years, and distance walked is very rarely in the normal range in any individual, supporting this as a reliable quantitative measure of therapeutic outcomes in clinical studies and for clinical monitoring.

## Methods

This study was an observational study open to any patient with a biochemically or molecularly confirmed diagnosis of BTHS as well as male controls who attended the Barth Syndrome International Conference in 2016. This study was approved under Johns Hopkins University IRB protocol “IRB00098987 Investigation into clinical, metabolic and molecular factors in Barth Syndrome.” Study enrollment took place in July 2016. Written informed consent was received from participants prior to inclusion in the study.

Participating individuals were males aged 6 to 34 who had molecular confirmation of BTHS or unaffected male controls age 6 to 30 years. Age, weight and height were measured and recorded for each participant.

### The 6 MWT

The 6 MWT was performed describing methods described in our 2016 paper [2], Briefly, the 6MWT was performed according to American Thoracic Society Guidelines, with the following exceptions made secondary to limitations of the testing location: the walking course was 50 ft (~ 15 m) as opposed to 30 m (100 ft) and the walk was performed on a carpeted surface [19]. All assessments were performed by physical therapists with familiarity with the 6MWT. All assessments were performed in bare feet to ensure consistency as participants arrived at the study wearing a wide variety of footwear. Paramedics were present for safety purposes and all subjects were independently ambulatory. Pre- and post- exercise fatigue was quantified via the Borg score [20]. 6MWT were compared both to the controls participating in this study, as well as to reported population norms for individuals aged 6-19y and > 20y.

### Muscle strength

All strength assessments were performed in subjects, with BTHS as well as controls, age 6 and up.

### Lower extremity muscle strength

#### Handheld Dynamometry

Knee extensor muscle strength was assessed by a single physical therapist with expertise in muscle strength measurements using protocol identical to that described in our previous paper published in 2016 [2]. Briefly, Knee extensors were chosen as the only muscle group to assess given noting that all lower extremity muscle groups are weak in children and adults with Barth Syndrome during our previous study. The average value of the 2 right knee extensor measurements and average value of the 2 left knee extensor measurements was used for data analysis. As in our previous study, the HHD strength data were assessed in 2 ways: normalized to body weight and raw to account for possible differences in body size. Results were compared to values from healthy controls obtained at the conference.

#### Grip Strength

Grip strength was measured by a single physical therapist with expertise in muscle strength measurements using published, standardized methodology via a JAMAR Hydraulic Hand Dynamometer. Grip strength was assessed utilizing techniques described in the NIH Toolbox and Jamar Hydraulic Handheld Dynamometer Owner’s Manual [21]. Participants were provided with a practice trial beginning with his dominant hand, followed by the non-dominant hand. The value from each single test trial for the right hand and left hand was utilized for data analysis. The grip strength data was assessed in two ways: normalized to weight and raw to account for possible differences in body size. Results were compared with control values obtained at the conference.

#### Five Times Sit to Stand Test

The 5 Times Sit to Stand Test (5 TSTS) is a functional assessment of lower extremity strength [22]. This test has been used to assess functional lower extremity strength in a variety of patient populations including: children with cerebral palsy [23], community-dwelling elderly [24], and patients with vestibular disorders [25]. The assessment was performed by a single physical therapist. Subjects all began in a chair with their arms folded across their chest with hips at approximately 90 degrees of flexion and knees at approximately 105 degrees of flexion in bare feet. For taller subjects, feet were positioned on the ground. For shorter subjects, feet were positioned on various height steps to obtain the desired starting position. Subjects were instructed to stand up and sit down as quickly as possible without stopping in between. A stopwatch was used to record the time in seconds and was stopped when the participant stood the 5th time. The value from single trial was used for data analysis. Results were compared with control values.

### Physical activity questionnaires

Physical activity questionnaires were administered using the same procedures as described in the previous study [2] to gain knowledge of general physical activity levels in subjects during their typical daily routine. Briefly, Subjects and parents of subjects 6–14 years of age completed the Physical Activity Questionnaire for Children (PAQ-C). The PAQ-C is a validated, self-administered, 7-day recall tool used to assess general levels of physical activity for children [26, 27]. The PAQ-C provides a summary physical activity score derived from nine items, each scored on a 5-point scale. A PAQ-C activity summary score of 1 denotes low physical activity, whereas a score of 5 denotes high physical activity [28, 29].

Subjects 15 years of age and older completed the International Physical Activity Questionnaire (IPAQ), Short Form, which is designed primarily for population surveillance of physical activity among adults (age 15–69 years). The IPAQ is a validated tool used to evaluate physical activity duration and vigorousness, in the prior 7 days.

### Balance

Balance was assessed in all subjects age 6 and up utilizing the SWAY Balance (SWAY Medical, Tulsa, Oklahoma) on a single iPhone (Apple commuter Inc., Cupertino, California) by 5 assessors trained in SWAY administration (4 assessors were physical therapists, 1 was a genetic counselor). SWAY Balance is an FDA-cleared balance testing system, which uses the built-in tri-axial accelerometers of a mobile electronic device to objectively assess postural movement [30]. The SWAY Balance sports protocol assesses: double limb stance eyes closed (EC), tandem stance right EC, tandem stance left EC, single leg stance right EC and single leg stance left EC while the subject holds the phone against his chest. The Sports protocol was used with the following modifications: test was performed in bare feet to ensure consistency of footwear between subjects and right and left single leg stance were performed with eyes open (EO) as opposed to eyes closed. We decided to complete the single leg stance portion with EO as opposed to EC as recommended by the application given that we were performing double limb stance and tandem stance with EC as well as the difficulty subjects with BTHS had during previous assessments performing single leg stance with EO. If the subjects were able, they completed at least 3 trials with the goal of a 95% confidence interval between the 3 trials as suggested by SWAY Balance [31]. The confidence interval is determined based on a color-coded system displayed on the phone screen. “Green indicates low variation of at least 3 tests. Yellow shows moderate variability with at least three baseline tests. Red indicates either high variability in a test score or not enough tests have been completed [31].” Data provided by SWAY Balance is on the SWAY scale from 0 to 100 with a higher score indicating better balance [30]. Data from the average of all trials completed was used in data analysis. Results were compared to control values obtained during the conference.

### Motion reaction time

Reaction time is a measure of sensory and neuromotor function that encompasses stimulus recognition and processing followed by the initiation of a neuromotor response [31]. SWAY Balance Motion Reaction Time Test was performed at the end of the Sports protocol by the same trained assessors that administered the Sports protocol. To perform the test, the subject was instructed to hold the iPhone in landscape with both hands on the device, press “Begin Test” to initiate the test and move the device in any direction when the screen turns orange for 5 trials [31]. Based on the 5 trials, an overall reaction time score is computed. “SWAY uses the raw measurement of reaction time, over five trials, to calculate an overall reaction time score for each test session. The fastest and slowest reaction time scores are dropped and an average of the remaining three scores is stored. A SWAY Score is derived from a proprietary algorithm to convert the reaction time score and variability to the 100-point Sway scale for comparison. Any score faster than 150 milliseconds, will convert to 100 on the Sway reaction Time scale [31].” Results of subjects with BTHS and controls were compared.

### Data analysis

Pearson r-correlations were determined for 6 MWT distance versus physical activity questionnaire responses and 6 MWT distance versus pre−/post exercise Borg fatigue scoring. R correlations were considered very strong if greater than or equal to 0.7, strong if between 0.4 and 0.69, moderate if between 0.3 and 0.39, weak if between 0.2 and 0.29, and none/negligible if between 0.01 and 0.19. 2-tailed student’s t-tests were used to determine the significant of grip strength, 6 MWT, HHD strength testing, and SWAY balance measurements between patients with BTHS and controls as well as between younger subjects (ages 4–19) and older subjects (ages 20–32).

Paired t-tests were performed to determine the relationship between the 2014 and 2016 data points for the 18 participants who participated in both studies including: 6 MWT distance, pre and post fatigue using the Borg scale, quadriceps strength assessed with HHD and physical activity measurements (Table 4).

**Table 4:** Comparison of height, weight, 6MWT, post- 6MWT fatigue minus Pre-6MWT fatigue for identical subjects with BTHS (n = 18) between 2014 and 2016. Fatigue assessed using the Modified Borg Scale

ID	Age 2014 (years)	Age 2016 (years)	Height 2014 cm (Z-Score)^a^	Height 2016 cm (Z-Score)^a^	Weight 2014 kg (Z-Score)^a^	Weight 2016 kg (Z-Score)^a^	6 MWT 2014 (m)	6 MWT 2016 (m)	Change (m)	Post-Pre Fatigue 2014	Post-Pre Fatigue 2016
1	6	8	112.4 (−1.33)	128 (−0.8)	20 (− 0.91)	27 (− 0.26)	313.9	276	−37.9	0	1
2	9	11	122.5 (−1.86)	135 (− 1.26)	19 (−3.54)	23 (−3.06)	436.8	360	−76.8	0.5	3
3	9	11	136 (−0.182)	147 (− 0.04)	24 (−1.7)	31 (− 1.32)	405.4	455	49.6	0.5	0
4	9	12	121.3 (−2.69)	128 (−3.6)	21 (− 2.99)	25 (−3.77)	411.5	325.7	−85.8	1	1
5	10	12	133 (−1.0)	142 (−1.06)	24 (−2.07)	28 (− 2.29)	457.2	405	−52.2	3	2.5
6	10	12	139.5 (−0.43)	149 (−0.71)	35 (− 0.08)	41 (− 0.43)	404.2	453	48.8	4	3.5
7	13	15	134.3 (−3.40)	140 (−2.74)	25 (− 4.62)	31 (− 3.00)	332.8	355.2	22.4	−2	−3
8	14	16	155 (−1.57)	173 (−0.26)	35 (−2.82)	42 (−2.96)	363.3	461	97.7	−0.5	0.5
9	16	18	163.6 (−1.38)	173 (−0.47)	37 (−3.68)	40 (−4.24)	345.3	376.1	30.8	2	3
10	17	19	172.8 (−0.41)	178 (0.18)	41 (−3.66)	47(− 3.02)	358.1	300.6	−57.5	0	0
11	19	21	175.5 (−0.16)	177	58 (−1.19)	62	536.4	560.2	23.8	0	2
12	20	23	168.8	168	52	59	403.6	392	−11.6	3.5	1
13	21	23	165.8	169	65	74	405.4	398.2	−7.2	2	3
14	21	24	180.8	183	66	76	315.5	386	70.5	1.5	7
15	24	26	165	165	44	45	407.8	354	−53.8	0	0
16	25	27	179.3	179	63	57	349	486	137	3	3
17	32	34	186.2	186	89	83	278	336	58	0	1
18	32	34	168.5	169	64	68	301.8	300	−1.8	0	0
